# Case Report: Graft *Versus* Tumor Effect After Non-Myeloablative Allogeneic Stem-Cell Transplantation in a Patient With Brentuximab-Vedotin Refractory Sezary Syndrome

**DOI:** 10.3389/fonc.2021.749691

**Published:** 2021-12-09

**Authors:** Georg-Nikolaus Franke, Konstantin Dumann, Madlen Jentzsch, Astrid Monecke, Christine Doehring, Claudia Nehring-Vucinic, Sebastian Schwind, Dietger Niederwieser, Uwe Platzbecker, Mirjana Ziemer, Vladan Vucinic

**Affiliations:** ^1^ Medical Clinic and Policlinic 1, Hematology, Cellular Therapy and Hemostaseology, University of Leipzig, Leipzig Medical Center, Leipzig, Germany; ^2^ Clinic for Dermatology, Leipzig Medical Center, Leipzig, Germany; ^3^ Institute for Pathology, Leipzig Medical Center, Leipzig, Germany; ^4^ Department for Hematology, Internal Oncology and Gastroenterology, Asklepios Hospital Weissenfels, Weissenfels, Germany

**Keywords:** allogeneic hematopietic stem cell transplantation, Sezary syndrome, brentuximab vedodin, graft vs leukemia effect, non-myeloablative conditioning

## Abstract

Sezary Syndrome (SS) is a rare leukemic variant of primary cutaneous T-cell lymphoma. Relapsed or refractory disease is generally considered incurable by conventional therapeutic approaches, although durable responses can be achieved with novel monoclonal antibodies. Allogeneic hematopoietic stem cell transplantation (alloHSCT) may have potential value by inducing graft vs-lymphoma (GvL) effects, but there is currently no consensus regarding the timing of alloHSCT or type of conditioning regimen. Here we present the case of a male patient who achieved a complete remission (CR) of primary refractory SS after non-myeloablative alloHSCT. Patient: Two years prior to HSCT, the patient had been refractory to CHOEP-based chemotherapy, interferon, extracorporeal photopheresis (ECP), and bexarotene. Directly prior to alloHSCT brentuximab-vedotin (BV) was applied resulting in a partial remission of the skin compartment and overall in a stable disease. Prior to HSCT, flow cytometry of the bone marrow and peripheral blood showed an infiltration with T-cells positive for CD5, CD4, low CD3, low CD2 and negative for CD7, CD38, HLA-DR and CD8. The trephine biopsy showed a 7% infiltration of SS cells. The CD4:CD8 ratio in peripheral blood (pb) was massively increased at 76.67, with 63.5% of white blood cells expressing a SS immune phenotype. The conditioning regimen included 30 mg/m2 fludarabine on days -5, -4 and -3 and total body irradiation with 2 Gy on day -1. Immunosuppression consisted of cyclosporine A from day-1 and mycophenolate mofetil from day 0. The patient received 6.55x106 CD34+ cells and 1.11x108 CD3+ cells/kg body weight. Bone marrow evaluation on day 28 still showed persistent SS cells by flow cytometry. After tapering immunosuppression until day 169, the CD4:CD8 ratio in pb normalized. CR was documented on day 169 after alloHSCT and is now ongoing for almost 3 years after alloHSCT. Conclusions: We confirm that an alloHSCT can be a curative option for refractory patients with SS. The achievement of a CR after tapering the immunosuppressive therapy indicates a significant role of the GvL effect. In present treatment algorithms for patients with SS, the timing of an alloHSCT and the intensity of conditioning should be further explored.

## Introduction

Primary cutaneous lymphomas are rare extramedullary lymphoproliferative diseases with primary presentation in the skin, but can also infiltrate lymph nodes, blood, and visceral organs (1, 2). The most common subtype is mycosis fungoides (MF). In early stages, MF is usually associated with indolent clinical course and favorable prognosis with survival of 10 – 35 years (3). The advanced stages of MF and Sézary syndrome (SS), a leukemic form of MF, presenting with erythroderma, Sézary cells in peripheral blood (pb) and lymphadenopathy usually associates with very poor prognosis and overall survival (OS) between 18% and 37% at 5 years (4). Sézary cells are expanded CD4+ cells with abnormal immunophenotype (CD4+/CD26- or CD4+/CD7-) (5). The expansion of CD4+ cells leads to an increased pb CD4:CD8 ratio, which can be used to monitor disease activity (6).

Conventional treatment options are limited to extracorporeal photopheresis (ECP), application of agents like bexarotene +/- interferon-alpha, psoralene and ultraviolet-A +/- interferon-alpha, chemotherapy, alemtuzumab, antibody conjugate directed against CD30 and C-chemokine receptor 4 (CCR4) and have unfortunately no curative potential.

An allogeneic hematopoetic stem cell transplantation (alloHSCT) remains the only potentially curative approach for patients with advanced SS that may offer long-term remissions, but also comes at the cost of higher treatment-related mortality. Due to comorbidities or advanced age (7), the majority of patients are not suitable for conventional myeloablative conditioning. Subsequently, reduced intensity or non-myeloablative conditioning (NMA) focusing primarily on engraftment and not on anti-tumor activity were developed (8, 9). Experiences of alloHSCT with NMA conditioning in SS patients are limited to small series (10, 11). Here we report on the graft-vs-Sézary effect in a primary refractory patient with SS who achieved long-term outcomes after alloHSCT with NMA conditioning.

## Patient

**Figure 1:**
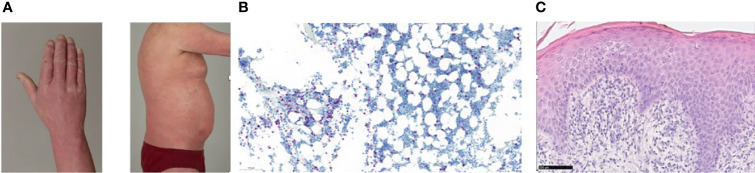
**(A)** Erythroderma of the skin prior to alloHSCT. **(B)** Trephine biopsy (CD3, 15x) with infiltrates of Sézary cells, **(C)** Skin histology (hematoxilin-eosin stain, 40x) showing diffuse infiltration of the papillary dermis with Sézary cells.

A 54-year-old Caucasian male patient was referred to our institution, initially with the diagnosis of a T-cell lymphoma, not otherwise specified, which was refractory to two courses of chemotherapy (CHOEP: cyclophosphamide, doxorubicine, vincristine, etoposide and prednisolone). At presentation an erythroderma involving >90% of the integument was predominant (**Figure 1A**). Computer tomography (CT) scans showed enlarged axillary, inguinal and cervical lymph nodes. The complete blood counts showed a leukocytosis of 24,300/µl. Flow cytometry of the pb revealed 11,664 Sézary cells/µl with CD4+CD7- phenotype and with a CD4:CD8 ratio of 85.5. Flow cytometry of the bone marrow aspirate confirmed CD30 positivity with expression of 7% in Sézary cells. Polymerase chain reaction of pb confirmed the clonality in T-cell receptor beta and gamma showing monoclonal Vβ-β2 and two clonal Vγ1-8-Jγ1.1 and 2.1 rearrangements. While conventional cytogenetics showed a normal male karyotype, fluorescent *in situ* hybridization (FISH) detected the deletion of chromosome 17p in 22 of 200 interphases with deletion of *TP53* gene. Immunohistochemistry of both trephine biopsy (**Figure 1B**) and skin histology revealed infiltrations with Sézary cells (**Figure 1C**). The skin histology also confirmed CD30 positivity with 5-10%, and the diagnosis was revised to SS.

The diagnosis was thus revised to Sézary Syndrome in Stage IVA (pT4 Nx M0 B2) according to the updated classification of International Society for Cutaneous Lymphomas (ISCL) and the European Organization of Research and Treatment (EORTC) (12).

Subsequently, successive therapies with 3,000,000 IU interferon alpha three times weekly for 6 months, combined with 10 mg/m^2^ methotrexate (MTX) and 19 courses ECP including bexarotene showed no improvement.

Six months prior to alloHSCT an antibody-conjugate treatment with brentuximab vedotin (BV) was administered. The patient received five courses, which led to an improvement of skin lesions and their reduction to about 30% of body surface. CT scans showed no further progression of the lymph nodes, the CD4:CD8 ratio was 76.7 with 63.5% Sézary cells in the flow-cytometry, confirming stable disease according to the EORTC and ISCL classification (4, 13).

Due to preexisting comorbidities, we performed an alloHSCT from an unrelated HLA identical (10/10) and cytomegaly virus matched (recipient and donor IgG positive) 30 year old male donor with non-myeloablative conditioning (fludarabine 30 mg/m² body surface area on d-4 to -2 and total body irradiation with 2 Gy on day -1) followed by infusion of pb stem cells (14). Immunosuppression consisted of cyclosporine A from d-1 (blood target level 200 ng/ml) and mycophenolate mofetil (3x1000 mg per day). The patient received a total of 6.6x10^6^ CD34+ cells/kg body weight, 1.1x10^8^ CD3+ cells/kg body weight and 0.2x10^8^ CD16+ cells/kg body weight. In the absence of acute graft-*versus*-host disease (GvHD), mycophenolate-mofetil was tapered 500 mg every 14 days from day 40 and discontinued on day 74 while cyclosporine A was tapered from day +56 and discontinued on d+196.

**Figure 2:**
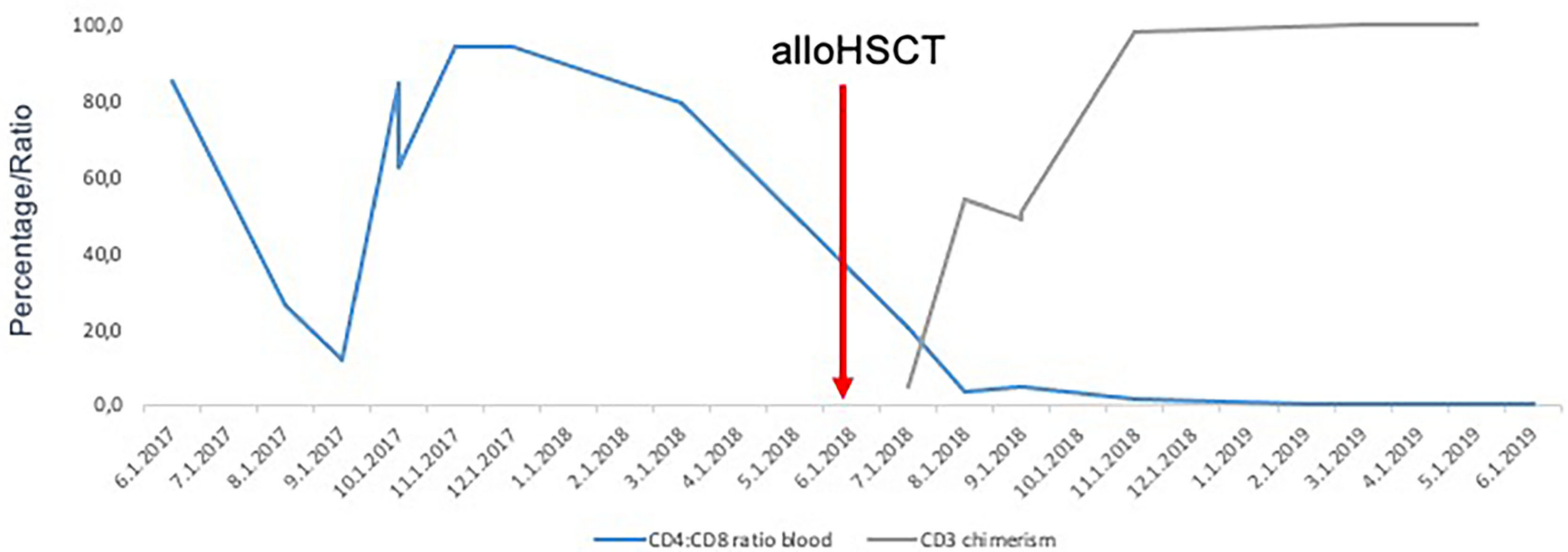
Normalization of CD4:CD8 ratio followed by increase of CD3+ donor chimerism after alloHSCT confirming graft-vs-Sézary effect.

The restaging on day +30 and +90 after alloHSCT showed residual infiltration of SS cells in the FACS analysis of the bone marrow (13 and 5% of all WBC, respectively) and chimerism of 70 and 93% on sorted CD3+ cells. The CT scan on day +90 after alloHSCT confirmed the persistent lymphadenopathy. On day + 172 after alloHCT a complete hematological remission (CR) with no infiltration of SS in bone marrow trephine biopsy, 100% chimerism on sorted CD3 positive cells from bone marrow aspirate and normal pb CD4:CD 8 ratio was documented, thus confirming graft-vs-Sézary effect (**Figure 2**). The CT scan showed no lymph node enlargements. The inspection of the skin revealed no suspect lesions.

After discontinuation of immunosuppression the patient developed chronic GvHD of the ocular mucosa and the liver according to national institutes of health (NIH) criteria (15) on day 277 and required immunosuppressive treatment with intermediate dosage of methylprednisolone, cyclosporine A and mycophenolic acid. The immunosuppression could have been slowly tapered to 720 mg mycophenolic acid b.i.d, 5 mg prednisolone and autologous plasma eye drops as previously described (16, 17). Furthermore, since September 2021 ruxolitinib was added to the GvHD treatment (18). Currently, 1215 days after alloHSCT the patient is in continuous complete remission of SS, having active ocular chronic GvHD.

## Discussion

We reported on a case of a 54-year-old male heavily pretreated caucasian patient with refractory stage IV SS with durable CR almost three years after alloHSCT following NMA conditioning, indicating a forceful graft-vs-Sézary effect.

AlloHSCT remains the only potentially curative option for patients with advanced SS, but the experience regarding alloHSCT is limited to a retrospective single-center analysis (19) and retrospective register analyses (20–22). Based on these data, the American Society of Bone Marrow Transplantation (ASBMT) published consensus guidelines on the use of alloHSCT in mature T-cell lymphomas advocating the use in relapsed or refractory SS patients (23). Both the European Society for Blood and Marrow Transplantation (EBMT) and German S2k guidelines for treatment of cutaneous lymphomas state alloSCT as an clinical option in stage IIB-IV (24, 25). Specific recommendations regarding conditioning regimen, graft source and optimal timing of the alloHSCT are lacking. In real-world setting alloHSCT is performed in prior heavily pretreated patients after exhausting prior treatment lines.

In 2010, Duarte et al. reported the EBMT register data of 60 patients with MF (n=36) and SS (n=24) undergoing alloHSCT (26) and updated them in 2014 with long-term follow-up (20). Most of the patients (n=70) had a sibling donor. RIC conditioning was applied in 44 patients and the source of stem cells was pb in 50 patients. Median OS at 5 and 7 years were 46% and 44%, respectively and median progression-free survival was 32% and 30%, respectively. In an updated analysis with an additional 73 patients Domingo-Domenech et al. (2021) reported a median overall survival and progression free survival (PFS) after 5 years of 38% and 26% respectively (21). The only independent adverse predictors for OS in multivariate analysis were advanced disease status and the use of an unrelated donor.

Lechowicz et al. reported (2014) retrospective data on 129 patients with MF and SS from Center for International Bone Marrow Transplant Research (CIBMTR) registry. 83 patients underwent alloHSCT after RIC or NMA conditioning regimens. The median OS after 5 years of the whole cohort was 32%, the median PFS after 5 years 17%. Non-relapse-mortality (NRM) after 5 years was 22%. The source of stem cells in most of the patients was PB.

Our patient underwent a NMA conditioning with fludarabine and 2 Gy TBI. Weng et al. recently (2020) reported a phase 2 trial using NMA conditioning regimen with total skin electron beam therapy, total lymphoid irradiation and anti-thymocyte globulin in 35 patients with MF (n=13) and SS (n=22), with a NRM of 14% after 2 years and a 5-year OS of 56% (10). The cumulative incidence of moderate/severe chronic GvHD after two years was 32%.

Prior to alloHSCT our patient was bridged with BV. Most of the Sézary cells show variable expression of CD30 positivity (27). Our patient showed positivity for CD30 both in trephine biopsy and in skin histology. BV, an antibody conjugate with selective microtubule disrupting agent directed against CD30 is a potential treatment option for SS patients. In an open-label, multicentric, phase III (ALCANZA) trial, BV was compared to standard treatment in patients with CD30 positive primary cutaneous T-cell lymphoma other than SS, showing significant improvement of objective responses after four months of treatment with 56.3% *versus* 12.5% respectively (28). Based on the results of this trial, BV was approved for treatment of European patients with CD30 positive primary cutaneous T-cell lymphoma after at least one previous therapy line.

While the experiences in T-cell lymphomas are limited, BV has been shown to be safe and effective as bridging to alloHSCT in patients with Hodgkin disease (29). Garciaz et al. (2019) reported on the treatment of 26 patients with T-cell lymphoma prior to alloHSCT, five of them with cutaneous T-cell lymphomas (30). BV did not show negative influence on engraftment or the GvHD rate.

Another potential treatment option for patients with SS is mogamulizumab, a humanized IgG1 CCR4 antibody, which is consistently expressed on tumor cells of patients with SS and MF (31). In a phase 3 open-label, randomized trial of mogamulizumab *versus* vorinostat in previously treated patients with CTCL (MAVORIC), mogamulizumab showed superior outcomes with median PFS of 7.7 months comparing to 3.1 in the vorinostat group.

AlloHSCT following NMA conditioning is a potentially curative option for patients with refractory SS. However, further evaluation is warranted to better define the best timing, most feasible conditioning intensity and graft source for the transplantation. Furthermore, as presented in this case we deem BV an adequate bridging to alloHSCT.

## Data Availability Statement

The raw data supporting the conclusions of this article will be made available by the authors, without undue reservation.

## Ethics Statement

Ethical review and approval was not required for the study on human participants in accordance with the local legislation and institutional requirements. The patients/participants provided their written informed consent to participate in this study. Written informed consent was obtained from the individual(s) for the publication of any potentially identifiable images or data included in this article.

## Author Contributions

G-NF, MZ, and VV wrote the manuscript. KD and AM provided histological evaluation of the skin and bone marrow. MJ, CN-V, CD, SS, DN, and UP provided administrative support. VV and MZ contributed equally and share the senior authorship. All authors contributed to the article and approved the submitted version.

## Conflict of Interest

G-NF, MJ, SS, UP, DN, KD, MZ, and VV receive honoraria from Takeda.

The remaining authors declare that the research was conducted in the absence of any commercial or financial relationships that could be construed as a potential conflict of interest.

## Publisher’s Note

All claims expressed in this article are solely those of the authors and do not necessarily represent those of their affiliated organizations, or those of the publisher, the editors and the reviewers. Any product that may be evaluated in this article, or claim that may be made by its manufacturer, is not guaranteed or endorsed by the publisher.
